# A distinct subpopulation of leukemia initiating cells in acute precursor B lymphoblastic leukemia: quiescent phenotype and unique transcriptomic profile

**DOI:** 10.3389/fonc.2022.972323

**Published:** 2022-09-21

**Authors:** Alex Q. Lee, Hiroaki Konishi, Connie Duong, Sakiko Yoshida, Ryan R. Davis, Jonathan E. Van Dyke, Masami Ijiri, Bridget McLaughlin, Kyoungmi Kim, Yueju Li, Laurel Beckett, Nitin Nitin, John D. McPherson, Clifford G. Tepper, Noriko Satake

**Affiliations:** ^1^ Department of Pediatrics, University of California (UC) Davis School of Medicine, Sacramento, CA, United States; ^2^ Genomics Shared Resource, University of California (UC) Davis Comprehensive Cancer Center, Sacramento, CA, United States; ^3^ Flow Cytometry Shared Resource, University of California (UC) Davis Comprehensive Cancer Center, Sacramento, CA, United States; ^4^ Department of Public Health Sciences, Division of Biostatistics, University of California (UC) Davis, Davis, CA, United States; ^5^ Departments of Food Science & Technology and Biological & Agricultural Engineering, University of California (UC) Davis, Davis, CA, United States; ^6^ Department of Biochemistry and Molecular Medicine, University of California (UC) Davis School of Medicine, Sacramento, CA, United States

**Keywords:** B-ALL, glucose, metabolic activity, leukemia initiating capacity, leukemia initiating cells, transcriptome profiling

## Abstract

In leukemia, a distinct subpopulation of cancer-initiating cells called leukemia stem cells (LSCs) is believed to drive population expansion and tumor growth. Failing to eliminate LSCs may result in disease relapse regardless of the amount of non-LSCs destroyed. The first step in targeting and eliminating LSCs is to identify and characterize them. Acute precursor B lymphoblastic leukemia (B-ALL) cells derived from patients were incubated with fluorescent glucose analog 2-(N-(7-Nitrobenz-2-oxa-1, 3-diazol-4-yl) Amino)-2-Deoxyglucose (NBDG) and sorted based on NBDG uptake. Cell subpopulations defined by glucose uptake were then serially transplanted into mice and evaluated for leukemia initiating capacity. Gene expression profiles of these cells were characterized using RNA-Sequencing (RNA-Seq). A distinct population of NBDG-low cells was identified in patient B-ALL samples. These cells are a small population (1.92% of the entire leukemia population), have lower HLA expression, and are smaller in size (4.0 to 7.0 μm) than the rest of the leukemia population. All mice transplanted with NBDG-low cells developed leukemia between 5 and 14 weeks, while those transplanted with NBDG-high cells did not develop leukemia (p ≤ 0.0001-0.002). Serial transplantation of the NBDG-low mouse model resulted in successful leukemia development. NBDG-medium (NBDG-med) populations also developed leukemia. Interestingly, comprehensive molecular characterization of NBDG-low and NBDG-med cells from patient-derived xenograft (PDX) models using RNA-Seq revealed a distinct profile of 2,162 differentially-expressed transcripts (DETs) (p<0.05) with 70.6% down-regulated in NBDG-low cells. Hierarchical clustering of DETs showed distinct segregation of NBDG-low from NBDG-med and NBDG-high groups with marked transcription expression alterations in the NBDG-low group consistent with cancer survival. In conclusion, A unique subpopulation of cells with low glucose uptake (NBDG-low) in B-ALL was discovered. These cells, despite their quiescence characteristics, once transplanted in mice, showed potent leukemia initiating capacity. Although NBDG-med cells also initiated leukemia, gene expression profiling revealed a distinct signature that clearly distinguishes NBDG-low cells from NBDG-med and the rest of the leukemia populations. These results suggest that NBDG-low cells may represent quiescent LSCs. These cells can be activated in the appropriate environment *in vivo*, showing leukemia initiating capacity. Our study provides insight into the biologic mechanisms of B-ALL initiation and survival.

## Introduction

Leukemia is a classic example of a disease of aberrant differentiation. Specifically, leukemia results from disrupted differentiation of pluripotent bone marrow progenitors into mature and functional mononuclear cells. Accordingly, leukemias are classified based on the intermediate stage at which differentiation is halted. In addition to lacking a terminally-differentiated phenotype and associated functional attributes, leukemia cells retain proliferative potential to continuously expand the population (1, 2). In some leukemias, as well as in most other cancers, it is now recognized that a rare but distinct subpopulation of cancer-initiating cells is responsible for population expansion and maintenance of tumor growth (3, 4). These leukemia stem cells (LSCs) are believed to make up an extremely small percentage of tumor cells, but they possess robust proliferative and self-renewal properties to facilitate sustained survival.

Despite the significance of LSCs in the development of curative leukemia treatments, definitive identification and characterization of LSCs in acute lymphoblastic leukemia (ALL) remains controversial (5, 6). While multiple leukemic subpopulations have been shown to be capable of propagating leukemia, their hierarchical structure is unclear or inconsistent. In addition, demonstration of leukemia initiating capacity alone is insufficient to validate LSCs, as it has been suggested that multiple ALL subpopulations with leukemia initiating capacity switch between dormancy and active proliferation, indicating a dynamic architecture of ALL development (7). As a result, it is very possible that ALL LSCs are by nature heterogeneous.

There are also few reliable distinguishing markers to characterize potential ALL LSCs. For example, hematopoietic stem cells (HSC) and LSCs in acute myeloid leukemia (AML) are traditionally defined based on phenotypic markers, including CD34+/CD38− (8–11). Characterization of these markers allows stem cells to be distinguished from their more differentiated progeny, which is a particularly crucial undertaking in the context of the heterogeneity of LSCs. In contrast, the phenotypic markers of stem cells in ALL, either B-cell or T-cell type, remain unclear. Previous studies demonstrated that subpopulations of ALL cells with CD34, CD38, CD20 or CD19 positive and/or negative markers could give rise to a leukemia phenotype in immunodeficient mice (12–14). These conflicting results demonstrate that bona fide phenotypic markers to distinguish leukemia initiating cells (LICs) in ALL are not yet available.

The metabolic activity of ALL is also poorly characterized. Previous reports have shown that ALL cells rely on glycolysis as their energy resource (the so-called Warburg effect) and an increased glycolysis rate was shown to be directly related to resistance to glucocorticoid treatment, which caused negative clinical outcomes (15–20). Thus, glycolysis is thought to have essential roles in ALL survival and malignancy. However, HSCs in general are known to be metabolically quiescent, with different manifestations in normal versus malignant HSCs (21–23). While normal HSCs have decreased oxidative respiration and instead rely on glycolysis, malignant HSCs–for example stem cells in AML–have been shown to possess lower levels of both glycolysis and oxidative respiration compared to non-stem cells in AML (24). Moreover, cancer stem cells (CSCs) are known to demonstrate metabolic plasticity, for example oscillating between oxidative phosphorylation and glycolysis to best promote tumor growth depending on the environmental state (25, 26).

In summary, there is quite significant inconsistency in characterizing the hierarchical structure, phenotypic markers, and metabolic activity of LSCs or CSCs. In this paper, we identified a unique subpopulation of B-ALL cells with leukemia initiating capacity within human patient xenografts and cell lines. These cells possess very low glucose uptake and a distinctive molecular profile relative to the broader cell population while also displaying potent leukemia initiating capacity *in vivo*. We hypothesize that these cells could represent LICs.

## Materials and methods

### Reagents

2-(N-(7-Nitrobenz-2-oxa-1, 3-diazol-4-yl) Amino)-2-Deoxyglucose (NBDG) was purchased from Invitrogen.

### Patient-derived leukemia cells

Primary patient leukemia samples were collected from patients with informed consent based on the UC Davis Institutional Review Board approved protocol and transplanted into 6–12-week-old female NOD/SCID/IL2Rg^-/-^ (NSG) mice using our institutionally-approved animal care protocol. As mice developed leukemia, they were sacrificed and leukemia cells were harvested from the leukemia-infiltrated spleen, bone marrow or liver for experiments. Human leukemia cells were confirmed by flow cytometry using anti-HLA-ABC and anti-CD10, CD19, CD22, CD34, CD38, CD45 antibodies were used for phenotyping (Biolegends & BD Biosciences).

### Cell isolation and sorting

Leukemia cells were resuspended in glucose-free DMEM and incubated with NBDG at 37°C in the dark for 30 minutes at 1ul of 5mg/ml NBDG per 3 million cells (27). After 30 minutes of incubation, leukemic cells were washed with PBS. The cells were then stained with anti-HLA antibody and 4’, 6-Diamidino-2-Phenylindole, Dilactate (DAPI) (Thermo Fisher Scientific). NBDG-low, NBDG-med, or NBDG-high cells were sorted by Cytopeia InFlux Cell Sorter (BD Biosciences) and BD Influx Cell Sorter (BD Biosciences) using 488nm excitation and 505-522nm emission wavelengths.

### Leukemia cell transplantation

The human leukemia mouse model was created by intratibial cell injection. The cell numbers transplanted ranged from 100 to 2.5 million cells per mouse. Sorted cells were used for some *in vivo* studies at the indicated cell doses. To determine the differentiation capacity of NBDG-low cells in xenograft mice, phenotypes of engrafted leukemia cells were compared with those of the original patient sample and/or serially-transplanted xenografts. Serial transplantations were established using the same technique and leukemia cells harvested from bone marrow, leukemia-infiltrated spleen, and liver (up to fourth generation mice).

Mice were monitored daily and euthanized when they showed signs of sickness, such as unkempt fur, ataxia, or weight loss more than 20% of pre-treatment body weight, in accordance with Institutional Animal Care and Use Committee (IACUC) policy on Humane Endpoints. Healthy mice were observed up to several months when they were euthanized to check whether they developed leukemia.

Statistical significance for survival time (days) was determined by the log-rank test. Kaplan-Meier survival curves were plotted for the two groups. Mice that did not develop human leukemia were censored. Two mice that had signs of sickness, but negative expression of HLA, were excluded from the study. Analyses used Prism 8.3 software (GraphPad).

### RNA-Sequencing library preparation and next-generation sequencing

Indexed, directional sequencing libraries were prepared directly from intact cells using the SMART-Seq Stranded Kit (Takara Bio, Inc.) according to the manufacturer’s standard protocol. Briefly, 300 cells were lysed and followed by random-primed first-strand cDNA synthesis, tailing, and template switching with reverse transcriptase (SMARTScribe RT). Illumina barcoded adapters were incorporated by PCR with SeqAmp DNA Polymerase and rRNA-derived cDNA was selectively depleted. The remaining mRNA-derived cDNA fragments were PCR-enriched and the resulting libraries purified with AMPure XP beads (Beckman Coulter, Indianapolis, IN). The RNA-Seq libraries were quantified using a Qubit dsDNA High Sensitivity Assay (Thermo Fisher Scientific) and sized by analysis with an Agilent 2100 Bioanalyzer (Agilent Technologies, Santa Clara, CA) using the Agilent High Sensitivity DNA Kit. Libraries were pooled and multiplex sequenced (2 x 150bp, paired-end) on an Illumina NovaSeq 6000 Sequencing System.

### RNA-Seq data analysis

De-multiplexed raw sequencing data (FASTQ format) was evaluated using FastQC (Babraham Bioinformatics) (28) and passed standard quality metrics, such as per base quality scores with ≥ 85% of bases >Q30. Additional NGS data quality control, pre-processing, alignment, quantification, differential expression, and statistical analysis were performed with Partek Flow Genomic Analysis software (Partek Inc., Chesterfield, MO). Sequence reads were pre-processed to trim 3’ adapter sequences and SMART-Seq Stranded library-specific sequence (i.e., first three bases of read two). Reads were then mapped to the GRCh38/hg38 human reference genome assembly using the STAR (Spliced Transcripts Alignment to a Reference; version 2.7.8a) (29) aligner. Transcript expression quantification was performed using the quantify to annotation model (Partek E/M) with GENCODE Release 36 (GRCh38.p13) annotation. Differential expression analysis (e.g., group-wise comparison of NBDG-low vs. NBDG-med) was performed with DESeq2 (30) using the poscounts estimation method. Hierarchical clustering of differentially-expressed transcripts (DETs) was performed using average linkage cluster and Euclidean point distance metrics. Functional annotation enrichment analysis was performed on DETs using the ToppFun tool in the ToppGene Suite (31). Benjamini-Hochberg’s FDRs or Storey’s q-values were used to correct for multiple testing.

## Results

### A distinct NBDG-low population was identified in B-ALL cell lines and patient samples

**Figure 1:**
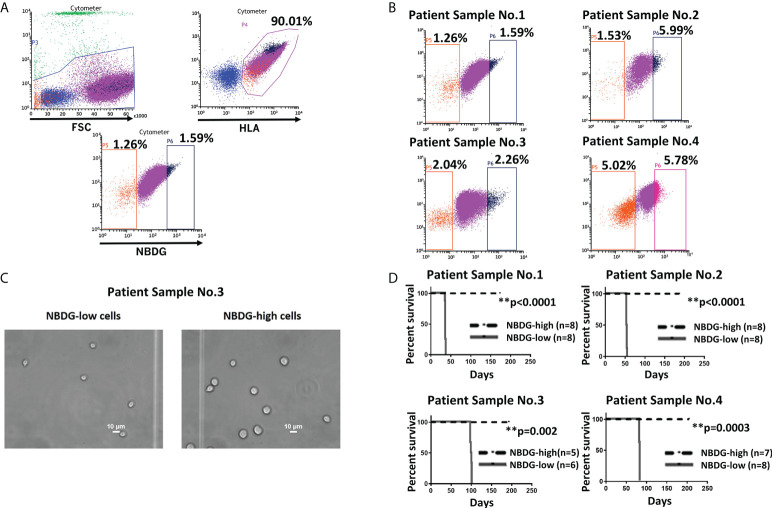
Identification of a distinct subset of B-ALL cells based on low glucose uptake and leukemia initiating capacity. **(A)** Gating strategy and sorting profile of patient-derived B-ALL cells by NBDG are shown. After gating DAPI-negative and HLA-positive cells, a distinct population was identified from the rest of the population by NBDG uptake levels. Low-uptake NBDG cells (NBDG-low cells, orange dots) are smaller, HLA-dim, and a small population. In contrast, high-uptake NBDG cells are bigger, HLA-strongly positive, and a majority of the population. Very high-uptake NBDG cells (NBDG-high cells) (black dots) were used as the counterpart of NBDG-low cells. **(B)** The distinct population of NBDG-low cells is detected in different patient samples. **(C)** Phase microscopy pictures of NBDG-low and NBDG-high cells show NBDG-low cells are smaller than NBDG-high cells (example results of PS3 are shown). **(D)** All mice that were injected with NBDG-low cells, using four patient samples (PS1-4), developed leukemia while none of NBDG-high groups developed leukemia (p ≤ 0.0001-0.002). In four patient samples (PS1-4), transplanted cells ranged between 5,000 and 50,000 cells per mouse based on the available sorted cells at the time (PS1: 10,000 cells in three mice and 50,000 cells in five mice. PS2: 5,000 cells in four mice and 10,000 cells in four mice. PS3:10,000 cells in six mice for NBDG-low and five mice for NBDG-high. PS4:10,000 cells in eight mice). ** means p<0.01.

To address the hypothesis that we could distinctly characterize B-ALL cells on the basis of metabolic features, we utilized the fluorescent glucose analog 2-(N-(7-Nitrobenz-2-oxa-1, 3-diazol-4-yl) Amino)-2-Deoxyglucose (NBDG) and distinguished low- and high-fluorescing cells with flow cytometry. These cells were referred to as NBDG-low (low glucose uptake) and NBDG-high (high glucose uptake) cells, respectively (32). A distinct population of NBDG-low cells was detected in the cell lines JM1 and Reh (**Supplementary Figure 1**) as well as in patient-derived B-ALL cells. The median rate of NBDG-low cell population was 1.92% (ranging from 0.36 to 5.75%) of the whole leukemia population, calculated by averaging the NBDG-low populations as a percentage of total leukemia cells, using 16 flow cytometry sorted samples (**Supplementary Table 1**). NBDG-low cells were smaller in size, ranging from 4.0 to 7.0 μm, compared to the counterpart NBDG-high cells, ranging from 5.0 to 14.0 μm (**Figures 1A-C**, and **Supplementary Figure 2A**). The NBDG-low cells also showed lower HLA expression than the NBDG-high cells (**Figure 1A**).

### NBDG-low cells isolated from patient samples exhibit potent *in vivo* leukemia initiating capacity

Leukemia initiating capacity for LICs is defined based on a transplantation assay, which tests their capacity to initiate, propagate, and maintain bulk leukemia growth *in vivo* as a xenograft in NSG mice. To confirm our hypothesis that NBDG-low cells have leukemia initiating capacity, we isolated NBDG-low or NBDG-high cells from six patient samples (PS) and transplanted the cells into NSG mice. Sample information is summarized in **Supplementary Table 2** (PS1-5 and PS10). All six patients were diagnosed with B-ALL; three patients were considered standard risk and three as high risk based on the currently used risk categorization (33). All the mice transplanted with NBDG-low cells developed leukemia between five and 14 weeks, whereas those transplanted with NBDG-high cells did not develop leukemia in the observed period, which was extended to more than three to four months after leukemia development in the NBDG-low group. The median survival time of the mice in the NBDG-low group was PS1: 36.0 days, PS2: 53.5 days, PS3: 98.5 days and PS4: 82.0 days. Mice in the NBDG-high groups of these four samples survived significantly longer than those of NBDG-low groups (p ≤ 0.0001-0.002) (**Figure 1D**).

The potency of NBDG-low cells for *in vivo* LIC was next examined by transplanting a very low number of cells (100 cells per mouse) using two samples, PS4 and PS5. In PS4, eight out of 14 mice transplanted with NBDG-low cells developed leukemia, whereas none of the 14 mice transplanted with NBDG-high cells developed leukemia by the end of the study. In PS5, six out of 11 mice transplanted with NBDG-low cells develop leukemia by the end of the study. However, none of the 12 mice transplanted with NBDG-high cells developed leukemia except that one mouse was found to have a partial leukemia phenotype at the end of the study. The median survival time of mice in the NBDG-low group is PS4: 263.5 days and PS5: 217.0 days. Mice in the NBDG-high groups of these two samples survived significantly longer than those in the NBDG-low groups (p=0.0009-0.02) (**Supplementary Figure 2B**).

**Figure 2:**
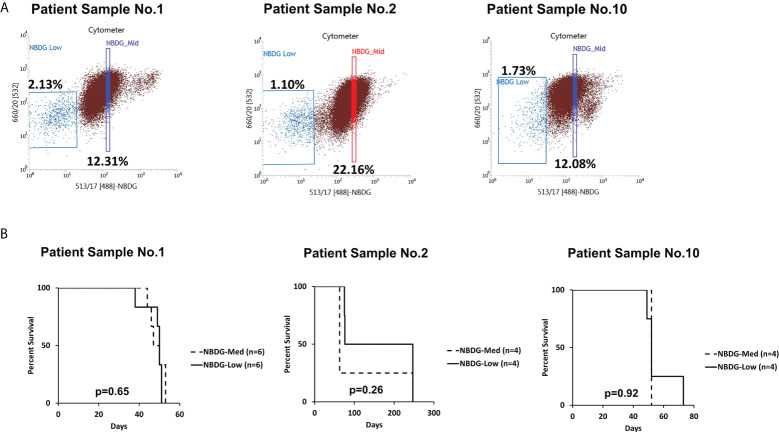
The NBDG-med population also initiates leukemia *in vivo*. **(A)** The NBDG-med population has intermediate NBDG uptake and lies in between the NBDG-low population and the NBDG-high population (not labeled; see Figure 1B). **(B)** In three separate experiments with the three patient samples described in **(A)**, mice that were transplanted with NBDG-med developed leukemia at a nearly identical rate to the NBDG-low mice, with overall median survival of 52 days for NBDG-med compared to 51.5 days for NBDG-low. 5000 cells were transplanted per mouse.

Of note, cells with an intermediate level of NBDG uptake (NBDG-med), located between the NBDG-low and NBDG-high populations (**Figure 2A**), from three patient samples (PS1, PS2, PS6) were transplanted and these mice subsequently also developed leukemia. Survival curves compared to NBDG-low transplanted mice are shown in **Figure 2B**. Combined median survival for the NBDG-med mice (n=14) from the three samples was 52 days, compared to 51.5 days for the NBDG-low mice (n=14). One mouse in the NBDG-med group and two mice in the NBDG-low group showed no signs of leukemia at the end of the experiment (247 days). These results indicate that NBDG-med cells were also able to establish an *in vivo* leukemia phenotype, similar to NBDG-low cells.

### NBDG-low cells show *in vivo* differentiation and self-renewal capacity

Overall, the leukemia developed from transplanted NBDG-low cells recapitulate the original B-ALL phenotypes (results of PS4 are shown in **Supplementary Table 3**). These results indicated that NBDG-low cells were able to reconstitute and reestablish the complete leukemic phenotype *in vivo*, similar to the original leukemia phenotype. We further confirmed the self-renewal capacity of the NBDG-low cells by serial transplantation of the PS1 xenograft model. All the serially-transplanted mice successfully developed leukemia (n=9, three independent experiments). Altogether, these results demonstrated the leukemia initiating capacity of NBDG-low cells in six different B-ALL xenografts (using five patient samples).

### NBDG-low cells have a unique transcriptome profile

As shown above, we identified a subpopulation of B-ALL cells from patient-derived xenograft (PDX) models that is characterized by low glucose uptake (i.e., based on NBDG fluorescence) and potent leukemia initiating capacity. To better understand the mechanisms underlying this biologic property, comprehensive molecular characterization was performed using next-generation RNA-sequencing (RNA-Seq)-based transcriptome analysis. This was performed on leukemia samples from five different B-ALL PDX models that were flow sorted into subpopulations based on NBDG fluorescence intensity, and therefore glucose uptake: NBDG-low (Low), NBDG-med (Medium), and NBDG-high (High). The RNA-Seq data was processed with a STAR-Partek(E/M)-DESeq2 analysis pipeline for read alignment, annotation, transcript expression estimation, and differential expression (DE) analysis as described in Materials and Methods.

**Figure 3:**
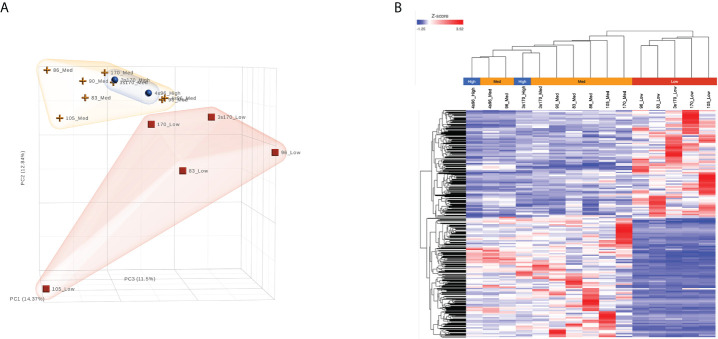
B-ALL LICs can be distinguished based on glucose uptake and molecular profile. RNA-Seq analysis was performed on five different human B-ALL PDX models flow sorted into subpopulations based on glucose uptake and NBDG fluorescence intensity: NBDG-low (Low), NBDG-med (Medium), and NBDG-high (High). Raw sequence data (FASTQ) were processed for read alignment, gene-level quantification, normalization, and differential expression as described in Materials and Methods. **(A)** Principal component analysis (PCA) performed on normalized read counts demonstrated organization of the samples into distinct groups of NBDG-low and a group comprised of NBDG-med and NBDG-high samples, as illustrated in the 3D PCA plot. **(B)** Differential expression analysis (DESeq2) identified a combined total of 2,512 DETs (p<0.05) in group comparison of NBDG-low samples to NBDG-med and NBDG-high samples. Hierarchical clustering and heat map visualization of the DETs (NBDG-low vs. NBDG-med, NBDG-low vs. NBDG-high, p<0.05) is presented and demonstrated distinct clustering of the NBDG-low samples and grouping of the NBDG-med and NBDG-high samples.

As shown in **Figure 3A**, unsupervised analysis of the normalized gene/transcript count data using principal component analysis (PCA) demonstrated that the data from the NBDG-low samples possessed sufficient variation from that of the NBDG-med and NBDG-high samples to distinguish them from the latter groups in the 3D plot and similarity in expression. Notably, the NBDG-med and NBDG-high uptake samples demonstrated tighter intra-group similarity of expression profiles while the NBDG-low sample group appeared to be more heterogeneous.

**Figure 4:**
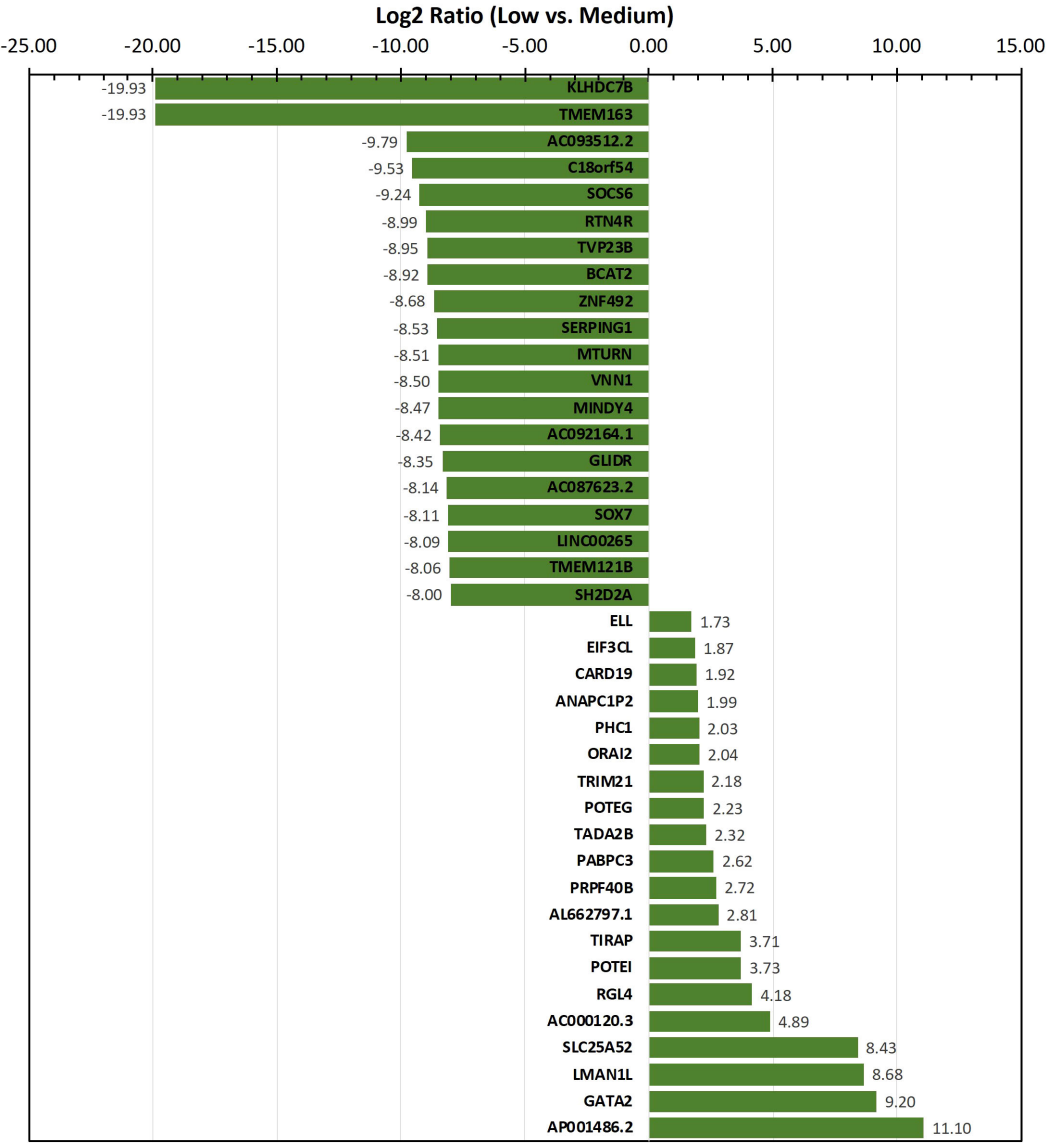
Transcripts exhibiting the highest level of differential expression in the NBDG-low population. Differentially-expressed transcripts (e.g., protein-coding genes, lncRNAs) in low glucose uptake B-ALL PDX subpopulations were identified as described above in **Figure 3** and Materials and Methods. Higher stringency statistical filtering was imposed (FDR p<0.05). Representative, differentially-expressed transcripts exhibiting the highest magnitude log2 ratios are presented in the bar chart with the corresponding values included as data labels.

To identify genes/transcripts whose altered expression were associated with and/or mediated the leukemia initiating phenotype, differential expression analysis was conducted by group-wise comparison of the NBDG-low and NBDG-med samples. The results revealed an expression profile of 2,162 DETs (NBDG-low vs. NBDG-med, p<0.05) consisting of 895 up-regulated and 1,267 down-regulated transcripts (**Supplementary Table 4**). Higher stringency statistical filtering resulted in 588 DETs (NBDG-low vs. NBDG-med, false discovery rate [FDR] p<0.05) (**Supplementary Table 5**) with a stronger bias towards down-regulated genes (n=424, 72.1%). Subsequent hierarchical clustering of these DETs and heatmap visualization demonstrated robust clustering into two major groups of samples: 1) NBDG-low and 2) NBDG-med with NBDG-high. In addition, there were two major patterns, and sub-clusters, of gene expression highlighted by marked uniformity in the expression of down-regulated genes in the NBDG-low samples and heterogeneous patterns of relative increased expression of genes in both the NBDG-med and NBDG-high samples (**Supplementary Figures 3A, B**). Similar group comparison of NBDG-low and NBDG-high samples revealed 1,104 DETs, with 656 and 448 being up- or down-regulated (p<0.05), respectively. When combined (i.e., “NBDG-low vs. NBDG-med” and “NBDG-low vs. NBDG-high” DETs), there were a total of 2,512 DETs, with 754 of these transcripts having commonly altered expression compared to that of both the NBDG-med and NBDG-high groups. In this instance, hierarchical clustering of the combined set of DETs and subsequent heatmap visualization demonstrated distinct segregation of the samples into 1) NBDG-low and 2) NBDG-med plus NBDG-high groups under two major branches of the dendrogram (**Figure 3B**). Additionally, two general clusters of increased and decreased gene/transcript expression were apparent for each group of samples, as well as the heterogeneity in up-regulated gene expression in each sample. Consistent with the striking leukemia-initiating phenotype of the NBDG-low subpopulations, these cells also exhibited a number of marked transcript expression alterations in both directions (e.g., log2 ratios of -19.93 and 11.10) (**Figure 4**; **Supplementary Tables 4, 5**).

**Figure 5:**
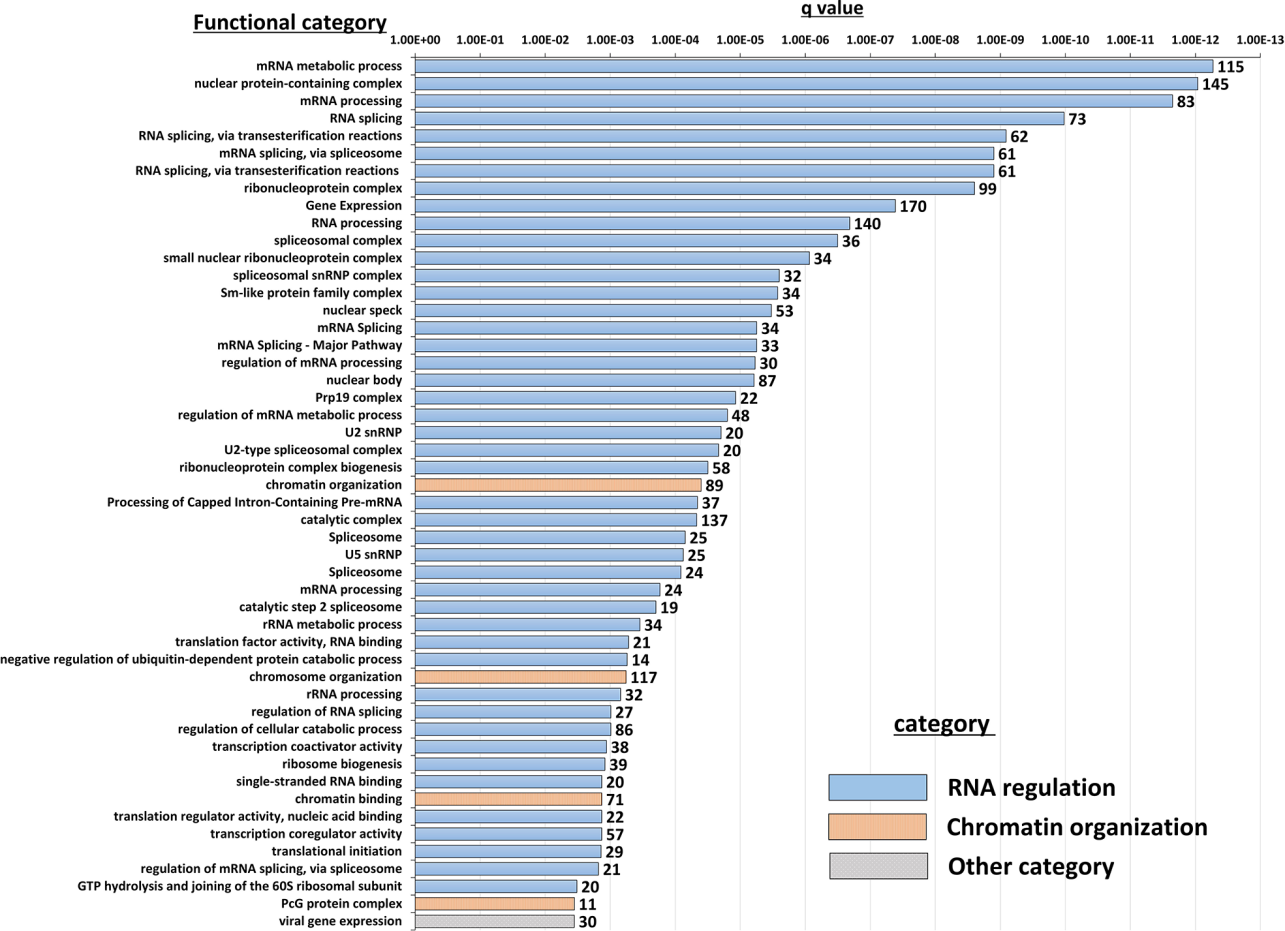
Functional annotation enrichment of the B-ALL NBDG-low expression signature. Differentially-expressed transcripts (p<0.05) in low glucose uptake B-ALL PDX subpopulations (i.e., relative to medium glucose uptake subpopulations) were identified as described above in **Figure 3** and Materials and Methods. Functional annotation enrichment analysis was then performed using ToppFun on the list of DETs as input. The enriched categories classified in GO; Molecular Functions, GO; Biological Process, GO; Cellular Component and Pathway were selected, and then the representative top 50 enriched functional categories and gene sets (y-axis) are displayed in the chart along with the Enrichment q-value (x-axis) and the number of gene hits from the NBDG-low expression profile in each category (data labels).

Insight into the potentially altered processes and pathways in the NBDG-low cells was gained by conducting functional annotation enrichment analysis of the DETs comprising the leukemia-initiating phenotype transcript profile using the ToppFun tool (31). The analysis yields over-represented genes in functional categories and gene sets from across multiple databases and collections, such as Gene Ontology Resource (34), KEGG (35), Reactome (36), and the Molecular Signatures Database (MSigDB) (37). The complete set of functional enrichment results for the NBDG-low transcript expression profile is provided as **Supplementary Table 6** and representative functional categories are presented in **Figure 5**. Notably, multiple functional categories of RNA metabolic processes, including RNA processing and splicing, followed by chromatin organization, are highly enriched in NBDG-low cells.

Furthermore, we investigated the metabolism-associated transcriptomic profile of the NBDG-low cells as these cells do not uptake glucose. Pathway analysis indicated that glucose metabolic steps, such as the TCA cycle and oxidative phosphorylation, are negatively regulated in NBDG-low cells. (**Supplementary Figure 6**).

## Discussion

LSCs are thought to be the root of cancer and are responsible for treatment resistance and disease relapse. However, LSCs have not yet been defined in B-ALL and no study that focuses on identifying B-ALL LSC populations by glucose metabolism has been reported yet. In this study, our group discovered a unique subpopulation of B-ALL cells by glucose uptake from primary B-ALL samples. We demonstrated that this cell population has *in vivo* leukemia initiating capacity and a distinct transcriptome profile. As detailed below, this population seems quiescent yet becomes activated in the appropriate *in vivo* environment, evidencing significant heterogeneity in leukemia initiation.

Our study showed that NBDG-low cells represent a small percentage of the entire leukemia population (median 1.92%) and are smaller in size (4.0 to 7.0 μm), which is consistent with phenotypes of quiescent stem cells (22, 38–43) **(** **Figure 1B**, **Figure 1C**, and **Supplementary Figure 2A**). The frequency of HSCs and AML LSCs has been reported to be 0.001-0.1% (44–48) and 0.00002-0.01% (10, 11), respectively. The frequency of CSCs, including those for colon cancer, neck tumors and breast cancer, has been reported to vary from 0.6 to 50%, depending on the type of cancer (49). Our finding of NBDG-low cells in the range 0.36 to 5.75%, with potentially only a fraction of these cells representing true cancer stem cells, seems to be more comparable to stem cells from hematopoietic origin.

There are two key functions that define stem cells: proliferation/differentiation and self-renewal (13, 14). The isolated NBDG-low cells consistently possess proliferative capacity in the six different series of PDX mouse models tested in this study (**Figure 1D**, **Figure 2B**, and **Supplementary Figure 2B**). The NBDG-low cells also retained the capacity to differentiate into leukemia phenotypically similar to that of the original patient leukemia as demonstrated by the expression of common cell surface markers, such as CD10 and CD19 (an example shown in **Supplementary Table 3**). Cell differentiation capacity, in that the NBDG-low cells form the same leukemia as the original type, was also demonstrated (an example shown in **Supplementary Table 3**). In addition, the self-renewal capacity of isolated NBDG-low cells was shown by successful serial transplantation of the isolated cells.

We additionally found that the NBDG-med population, similar to NBDG-low, has *in vivo* leukemia initiating capacity. As NBDG-low cells are quiescent yet possess leukemia initiating capacity, we speculate that these cells become activated into the NBDG-med population *in vivo*. This population may then terminally differentiate into the NBDG-high population and lose its leukemia initiating capacity, as well as potentially de-differentiate back into the NBDG-low population. This notion is supported by our transcriptome profiling showing that NBDG-low cells have a distinct profile, whereas the NBDG-med and NBDG-high populations cluster together (**Figure 3B**) (13). NBDG-low cells also cluster separately from NBDG-high and unsorted (Whole) cell populations using high throughput quantitative RT-PCR analysis (**Supplementary Figure 4**), establishing the NBDG-low profile as distinct from all other populations. Moreover, *in vitro* colony forming assays were performed using Reh and JM1 B-ALL cell lines, resulting in colony formation between 6-36 days in NBDG-low cells versus 5-25 days in NBDG-high cells (**Supplementary Figure 5**). The delayed colony formation observed in NBDG-low cells further supports the quiescent behavior of NBDG-low prior to activation and differentiation. Altogether, our findings suggest that leukemia initiation is a highly fluid process that involves requisite circumstances for activation and the capacity for continuous re- and de-differentiation.

Pathway analysis of glucose metabolism shows negatively regulated glucose metabolism process in NBDG-low cells (**Supplementary Figure 6**), which is consistent with the low glucose uptake phenotype of these cells. Of note, part of fat metabolism is upregulated in the NBDG-low cells, suggesting these cells may use fat for their energy source. Previous studies show that lipid metabolism is closely associated with maintenance of stemness in cancer stem cells (50, 51), indicating that LICs also utilize the lipidic metabolism pathway to maintain their properties, such as stemness and non-glucose dependency.

Interestingly, functional enrichment results show significant enrichment of genes associated with RNA processing, including mRNA splicing *via* spliceosome and the spliceosomal complex (**Figure 5**). Previous studies indicated that cancer cells utilize alternative splicing for their survival (52, 53). Interestingly, RNA processing alterations are common in the five NBDG-low samples, whereas PCA analysis of their transcriptome profiles are heterogeneous (**Figure 3**). This suggests that RNA processing changes commonly occur in NBDG-low cells affecting a variety of other pathways contributing to the heterogeneity of NBDG-low cells. Mutations in splicing have been shown to contribute to aberrant hematopoiesis and may be among the first mutations to occur in HSCs (54, 55). Recent studies have shown aberrant splicing to be highly prevalent in leukemia driver genes and to significantly contribute to leukemia pathogenesis in both AML and B-ALL (56, 57). In fact, splicing can also represent a critical target for future investigations on potential treatments and drug resistance. For example, spliceosome inhibitors including isoginkgetin and pladienolide B have been shown to be cytotoxic in both mouse and human tumor cell lines (58), supporting future *in vivo* studies on splicing inhibition in leukemia. Moreover, alteration of CD19 splicing and resultant impairment of recognition by CD19-specific T-cells has been shown to contribute to treatment resistance (59); our study provides a possible genetic explanation for this finding. These suggest that NBDG-low cells modify the transcribed RNAs through the RNA binding proteins as well as the gene expression by the formation of heterochromatin to maintain their quiescent and undifferentiated phenotype. Furthermore, there is enrichment of genes associated with protein translation in the NBDG-low group; this is consistent with the known effects of aberrant translation on the pathogenesis of cancer (60, 61). For example, assembly of the eukaryotic initiation factor 4F complex has been shown to be essential for translation of mRNAs that contribute to cell growth and inhibition of apoptosis (62). This represents an additional genetic factor potentially driving the leukemia initiating properties of the NBDG-low population, as well as a therapeutic target for future studies.

Genes regulating chromosome organization, such as *SMC1*, *TP53*, and *PARP1* are also up-regulated in the NBDG-low, leukemia-initiating subpopulation (**Supplementary Table 6**). These genes have been reported to be associated with cell cycle regulation and maintenance of stem cell quiescence (63–65). Histone deacetylases (HDACs) play a major role in epigenetic gene regulation *via* histone modification and chromatin remodeling, and they maintain LSC survival in chronic myeloid leukemia (CML) and AML (66–69). *HDAC5*, up-regulated in the NBDG-low population, may lead to the maintenance of quiescence in leukemia initiating cells.

In summary, our findings demonstrate the robustness of utilizing NBDG to consistently and accurately identify a unique subpopulation of leukemia-initiating B-ALL cells based on glucose uptake. This suggests the future potential for using other indicators of metabolic activity to develop a more comprehensive characterization of LICs. Importantly, gene expression profiling of multiple B-ALL PDXs demonstrated that the NBDG-low population can be clearly distinguished from the NBDG-med, NBDG-high, and whole cell populations by the differential expression of a distinct set of NBDG-low signature genes. Therefore, the NBDG-low gene expression signature provides further insight into the biological mechanisms underlying their persistence and recurrence and represents a molecular tool for identifying a distinct subset of LICs in B-cell ALL PDXs.

## Data availability statement

The datasets presented in this study can be found online in the National Center for Biotechnology Information Gene Expression Omnibus (NCBI GEO) repository with the accession number GSE206258.

## Ethics statement

The studies involving human participants were reviewed and approved by UC Davis Institutional Review Board. Written informed consent to participate in this study was provided by the participants’ legal guardian/next of kin. The animal study was reviewed and approved by UC Davis Institutional Animal Care and Use Committee.

## Author contributions

NN, CT and NS contributed to the conception and design of the study. AL, CD, SY, and MI conducted the mouse experiments. JD and BM assisted with cell sorting. HK, RD, JM, and CT performed the gene expression profiling and analyses. KK, YL and LB performed the statistical analysis. AL, HK, MI, and CT wrote sections of the manuscript. All authors contributed to the article and approved the submitted version

## Funding

This work was supported by research funding from the Keaton’s Child Cancer Alliance, The Hartwell Foundation, Hyundai Hope on Wheels, Academic Senate Research Grant, Children’s Miracle Network Grant, Cancer Research Coordinating Committee Grant, National Center for Advancing Translational Sciences, NIH through Grant #UL1 TR000002, CTSC-MCRTP, Mr. and Mrs. George Davis, Mr. and Mrs. Ray Diamondstone, and Salad Cosmo (Satake), the Walter and Loretta Tepper Memorial Cancer Research Fund (Tepper), and the St. Baldrick’s Foundation and American Society of Hematology (Lee). The UC Davis Comprehensive Cancer Center Genomics Shared Resource, Flow Cytometry Shared Resource and Biostatistics Shared Resource are supported by Cancer Center Support Grant (P30CA093373) from the National Cancer Institute.

## Acknowledgments

The authors wish to thank Ms. Stephenie Y. Liu (Department of Pathology and Laboratory Medicine, Genomics Shared Resource, UC Davis Comprehensive Cancer Center) for her expert technical assistance for RNA sample processing.

## Conflict of interest

The authors declare that the research was conducted in the absence of any commercial or financial relationships that could be construed as a potential conflict of interest.

## Publisher’s note

All claims expressed in this article are solely those of the authors and do not necessarily represent those of their affiliated organizations, or those of the publisher, the editors and the reviewers. Any product that may be evaluated in this article, or claim that may be made by its manufacturer, is not guaranteed or endorsed by the publisher.
