# Supplementary material for: A distinct subpopulation of leukemia initiating cells in acute precursor B lymphoblastic leukemia: quiescent phenotype and unique transcriptomic profile
Source: Front Oncol. 2022 Sep 21;12:972323. doi: 10.3389/fonc.2022.972323 (PMC9533407; doi:10.3389/fonc.2022.972323)
Supplement: Supplementary file 2 [file Presentation_1.pptx]

## Slide 1
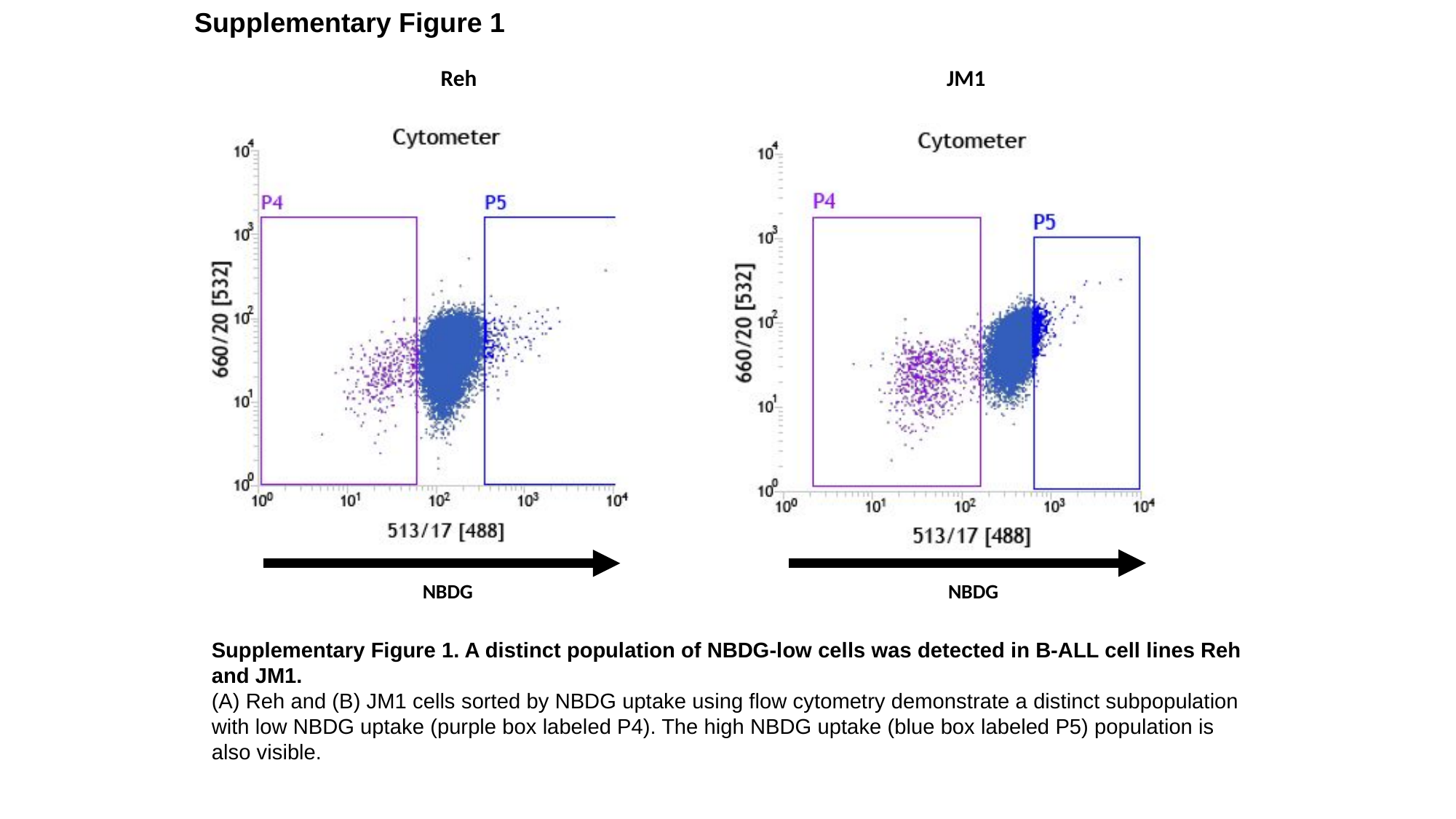

Supplementary Figure 1
Reh
JM1
NBDG
NBDG
Supplementary Figure 1. A distinct population of NBDG-low cells was detected in B-ALL cell lines Reh and JM1.
(A) Reh and (B) JM1 cells sorted by NBDG uptake using flow cytometry demonstrate a distinct subpopulation with low NBDG uptake (purple box labeled P4). The high NBDG uptake (blue box labeled P5) population is also visible.

## Slide 2
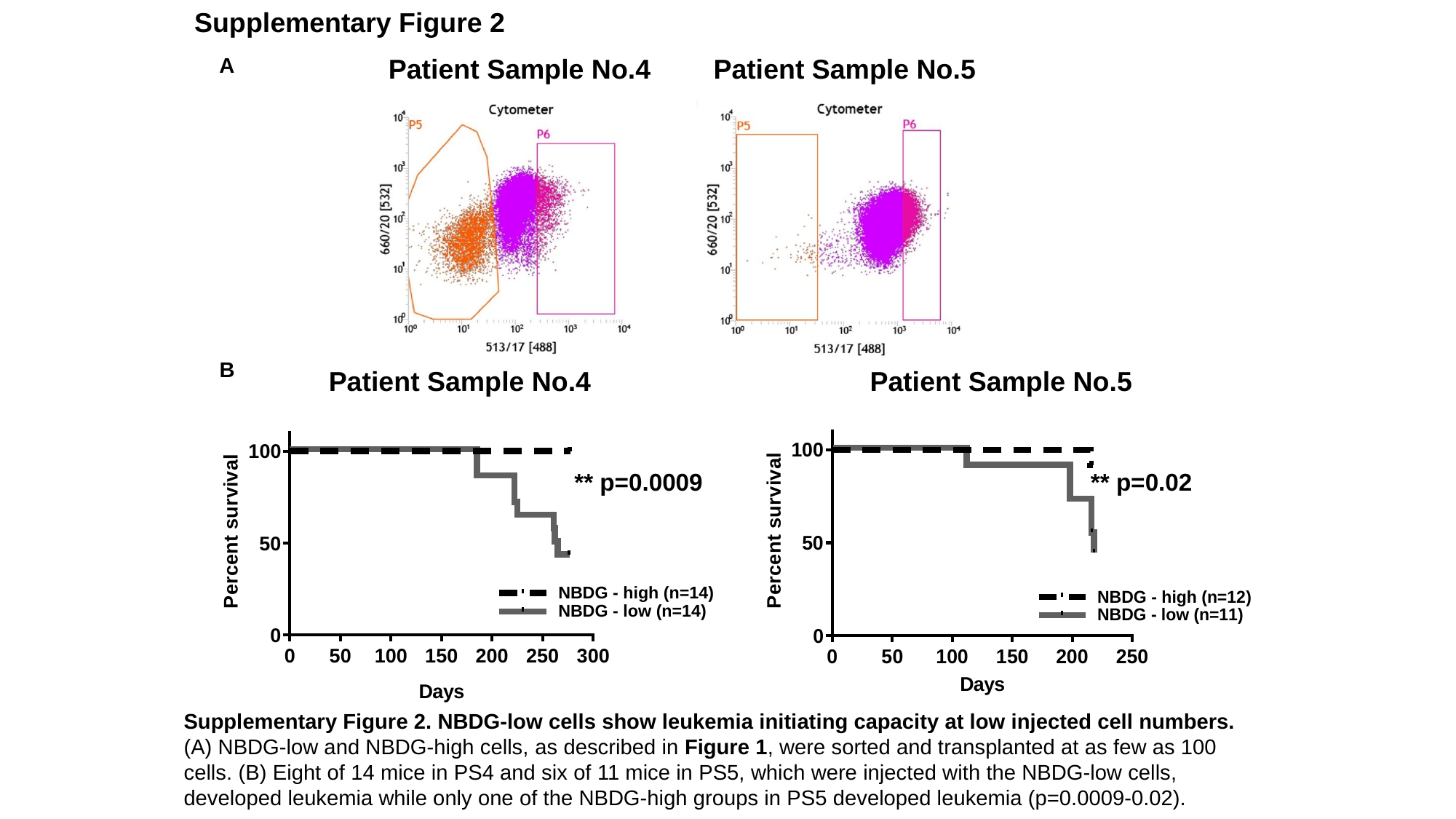

Supplementary Figure 2
Patient Sample No.4
Patient Sample No.5
A
B
Patient Sample No.4
Patient Sample No.5
** p=0.0009
** p=0.02
Supplementary Figure 2. NBDG-low cells show leukemia initiating capacity at low injected cell numbers.
(A) NBDG-low and NBDG-high cells, as described in Figure 1, were sorted and transplanted at as few as 100 cells. (B) Eight of 14 mice in PS4 and six of 11 mice in PS5, which were injected with the NBDG-low cells, developed leukemia while only one of the NBDG-high groups in PS5 developed leukemia (p=0.0009-0.02).

## Slide 3
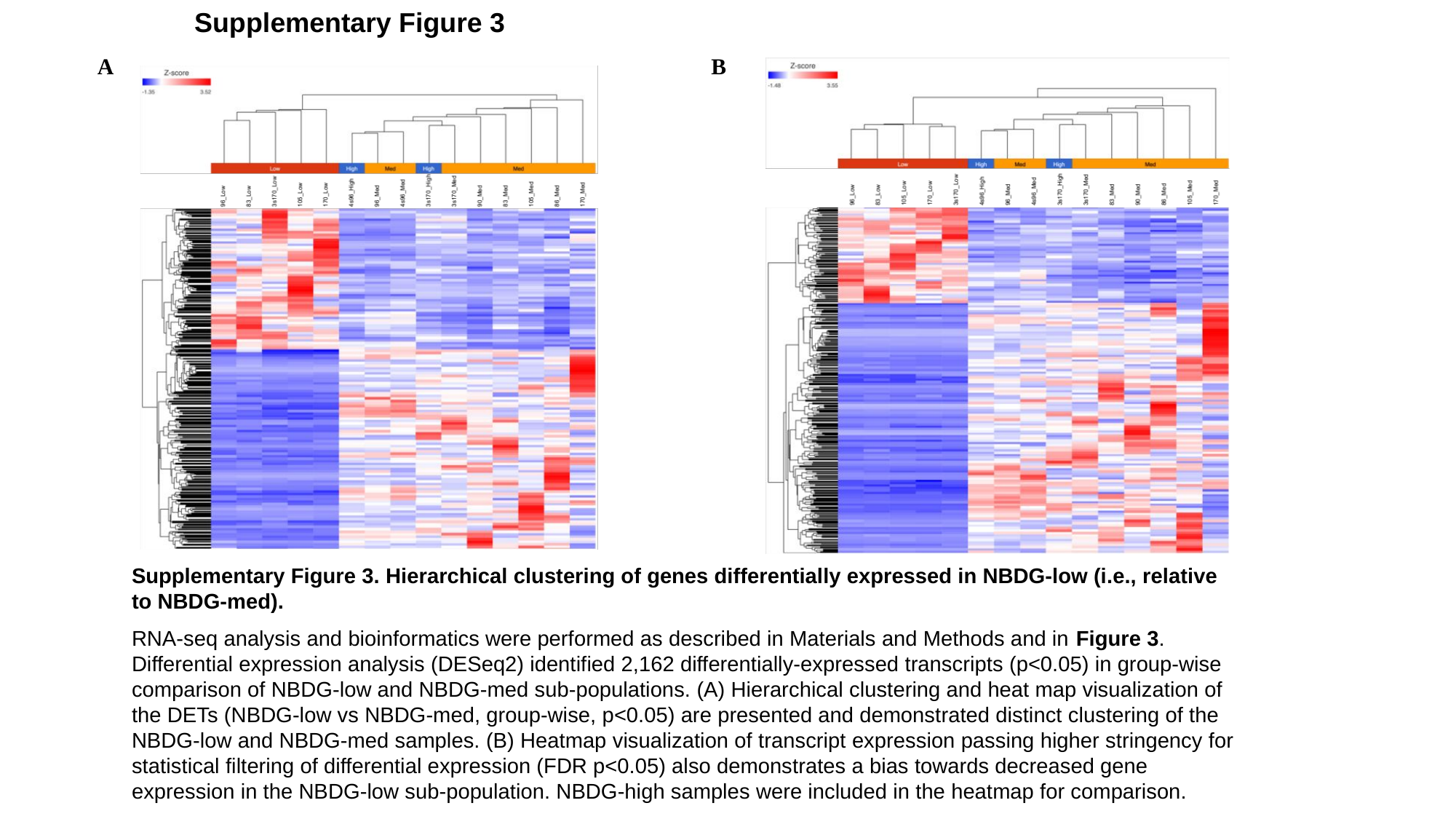

Supplementary Figure 3
B
A
Supplementary Figure 3. Hierarchical clustering of genes differentially expressed in NBDG-low (i.e., relative to NBDG-med).
RNA-seq analysis and bioinformatics were performed as described in Materials and Methods and in Figure 3. Differential expression analysis (DESeq2) identified 2,162 differentially-expressed transcripts (p<0.05) in group-wise comparison of NBDG-low and NBDG-med sub-populations. (A) Hierarchical clustering and heat map visualization of the DETs (NBDG-low vs NBDG-med, group-wise, p<0.05) are presented and demonstrated distinct clustering of the NBDG-low and NBDG-med samples. (B) Heatmap visualization of transcript expression passing higher stringency for statistical filtering of differential expression (FDR p<0.05) also demonstrates a bias towards decreased gene expression in the NBDG-low sub-population. NBDG-high samples were included in the heatmap for comparison.

## Slide 4
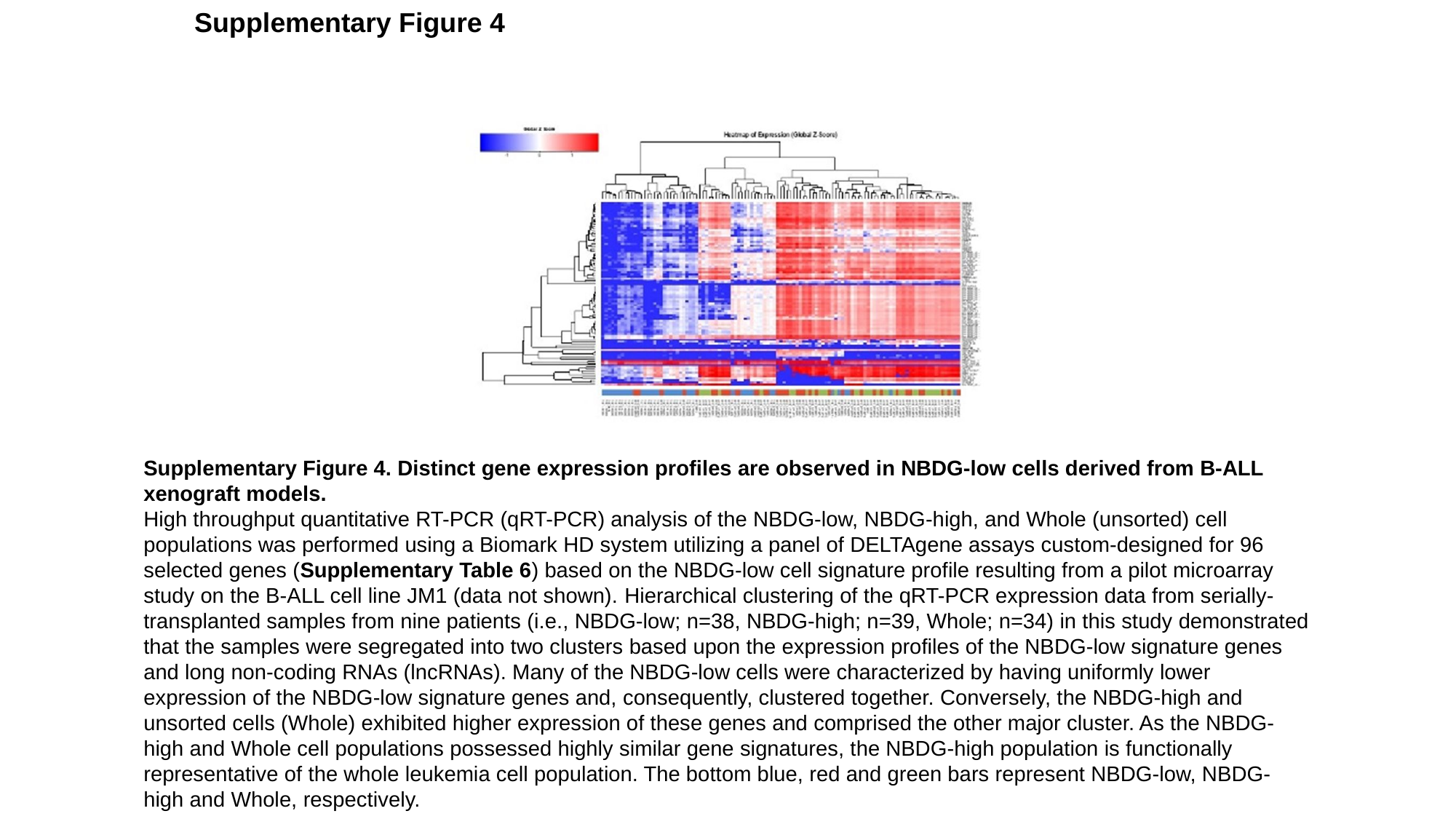

Supplementary Figure 4
Supplementary Figure 4. Distinct gene expression profiles are observed in NBDG-low cells derived from B-ALL xenograft models.
High throughput quantitative RT-PCR (qRT-PCR) analysis of the NBDG-low, NBDG-high, and Whole (unsorted) cell populations was performed using a Biomark HD system utilizing a panel of DELTAgene assays custom-designed for 96 selected genes (Supplementary Table 6) based on the NBDG-low cell signature profile resulting from a pilot microarray study on the B-ALL cell line JM1 (data not shown). Hierarchical clustering of the qRT-PCR expression data from serially-transplanted samples from nine patients (i.e., NBDG-low; n=38, NBDG-high; n=39, Whole; n=34) in this study demonstrated that the samples were segregated into two clusters based upon the expression profiles of the NBDG-low signature genes and long non-coding RNAs (lncRNAs). Many of the NBDG-low cells were characterized by having uniformly lower expression of the NBDG-low signature genes and, consequently, clustered together. Conversely, the NBDG-high and unsorted cells (Whole) exhibited higher expression of these genes and comprised the other major cluster. As the NBDG-high and Whole cell populations possessed highly similar gene signatures, the NBDG-high population is functionally representative of the whole leukemia cell population. The bottom blue, red and green bars represent NBDG-low, NBDG-high and Whole, respectively.

## Slide 5
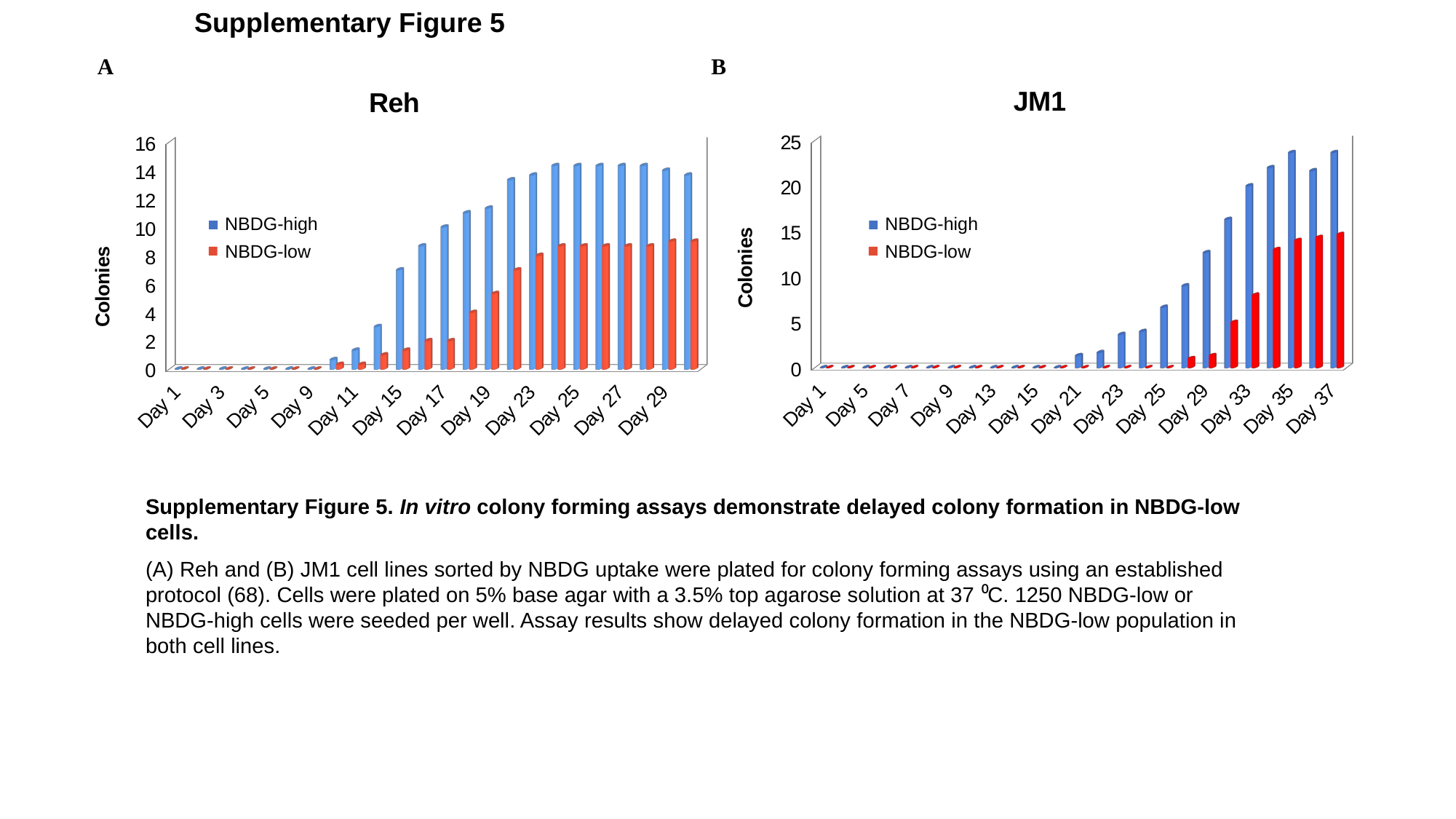

Supplementary Figure 5
B
A
[unsupported chart]
[unsupported chart]
NBDG-high
NBDG-low
NBDG-high
NBDG-low
Supplementary Figure 5. In vitro colony forming assays demonstrate delayed colony formation in NBDG-low cells.
(A) Reh and (B) JM1 cell lines sorted by NBDG uptake were plated for colony forming assays using an established protocol (68). Cells were plated on 5% base agar with a 3.5% top agarose solution at 37 ⁰C. 1250 NBDG-low or NBDG-high cells were seeded per well. Assay results show delayed colony formation in the NBDG-low population in both cell lines.

## Slide 6
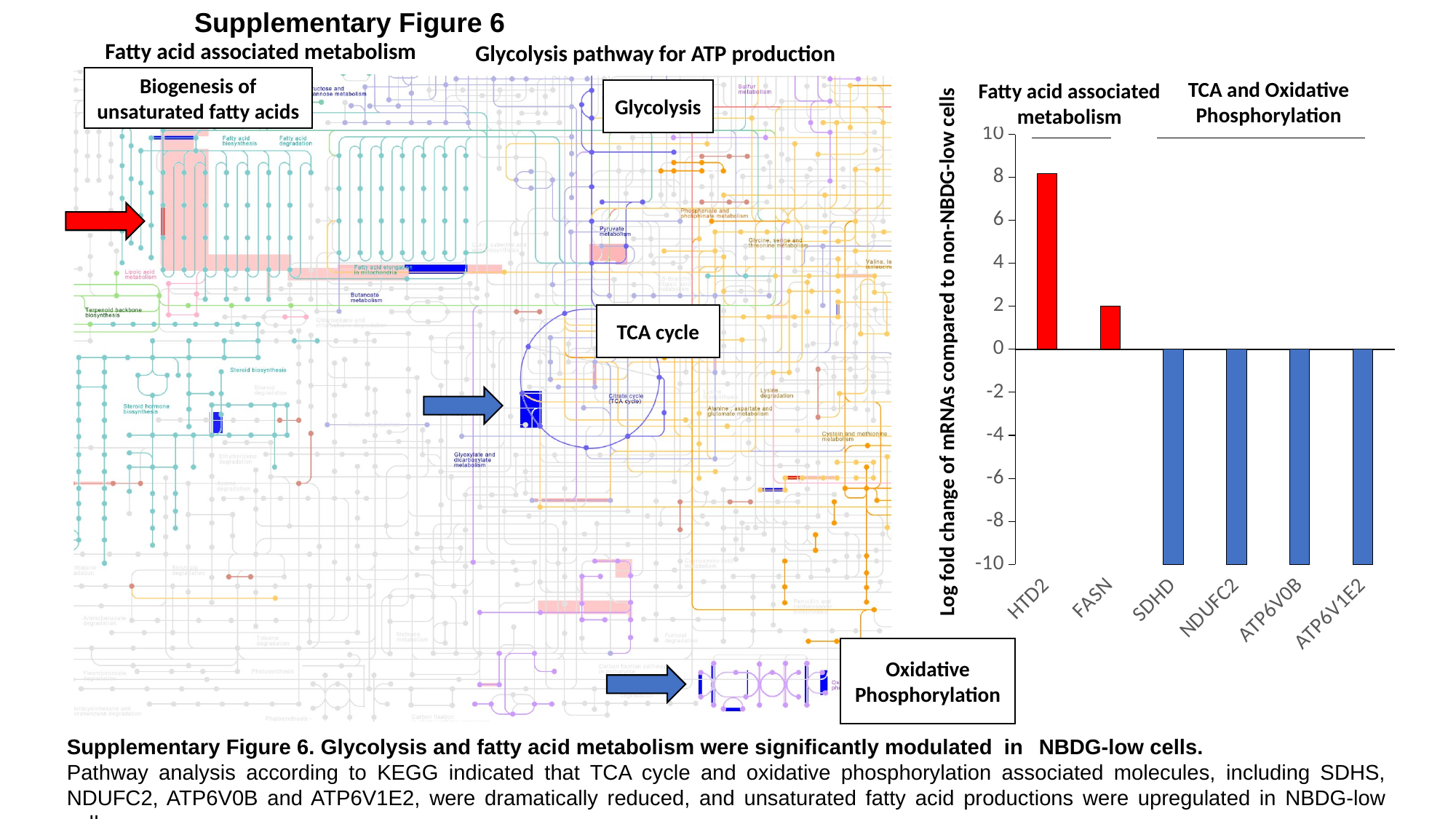

Supplementary Figure 6
Fatty acid associated metabolism
Glycolysis pathway for ATP production
Biogenesis of unsaturated fatty acids
TCA and Oxidative Phosphorylation
Glycolysis
Fatty acid associated metabolism
### Chart
| Category | |
|---|---|
| HTD2 | 8.1699 |
| FASN | 2.0147 |
| SDHD | -10.0 |
| NDUFC2 | -10.0 |
| ATP6V0B | -10.0 |
| ATP6V1E2 | -10.0 |
TCA cycle
Log fold change of mRNAs compared to non-NBDG-low cells
Oxidative Phosphorylation
Supplementary Figure 6. Glycolysis and fatty acid metabolism were significantly modulated in   NBDG-low cells.
Pathway analysis according to KEGG indicated that TCA cycle and oxidative phosphorylation associated molecules, including SDHS, NDUFC2, ATP6V0B and ATP6V1E2, were dramatically reduced, and unsaturated fatty acid productions were upregulated in NBDG-low cells.
